# Response to: Comment on “Complete Androgen Insensitivity Syndrome: Optimizing Diagnosis and Management”

**DOI:** 10.1155/2014/808270

**Published:** 2014-12-11

**Authors:** Antonio Simone Laganà, Alfonsa Pizzo

**Affiliations:** Department of Pediatric, Gynecological, Microbiological and Biomedical Sciences, University of Messina, Via C. Valeria 1, 98125 Messina, Italy

We appreciate Balsamo and colleagues' interest [1] in our recent paper [2] about a case of complete androgen insensitivity syndrome (CAIS). We are grateful for their insightful comments and the opportunity to clarify a number of elements from our work. Since many of the opinions expressed in their letter are based on biased interpretation of data, we would like to point-to-point answer their queries as follows.

1. We evidenced that the reported girl showed “normal intellectual function,” just as an anamnestic information for the differential diagnosis with other syndromes. It certainly was not our intention to express any opinion on the level of intelligence of patients suffering from CAIS.
2. As it is easy to evidence in the “Subject and Method” section, “Clinical Features” subsection of our paper, “The patient was sent to our observation by a provincial ambulatory service of gynecology and obstetrics, with a diagnosis of hypergonadotropic hypogonadism.” It is crystal clear that the presumed diagnosis of hypergonadotropic hypogonadism did not come from us and, in fact, we did not confirm it. Furthermore, as evidenced by Melo et al. [3], it is possible to have low levels of testosterone and elevated levels of follicle stimulating hormone (FSH) in CAIS patients with abdominal testes. Moreover, confirming the data of Melo et al. [3] based on postpubertal women with CAIS, estradiol level was not elevated (Melo et al.'s data: less than 10 and 40 pg/mL); luteinizing hormone (LH) was elevated (Melo et al.'s data: 14–43 IU/liter). As far as sex hormone binding globulin (SHBG) is concerned, it is reported by Audi and colleagues [4] that it could be very low in AIS patients (in particular, refer to CAIS patient number 5 and PAIS patient number 11 of Audi et al.'s work). Considering all these evidences from the works cited by Balsamo et al. [1], it is very easy to verify that the hormonal status of our reported patient is fully compatible with CAIS diagnosis.
3. In the work by Melo et al. [3], seven postpubertal women with CAIS with intact testes began breast development at the age of 11–15 years. Our work [2] reported a case of a 16-year-old girl, so it is possible that the breast characteristic, described as “hypotrophic,” may be due to the early stage of development of this secondary sexual feature. Moreover, the age in which breast development starts has not been studied extensively but has been reported as delayed in some individuals [5].
4. As already evidenced above (point number 2 of this reply), the patient came to our observation with a diagnosis of hypergonadotropic hypogonadism, established by others than authors. Following this diagnosis, we decided to administrate hormone replacement therapy with oral estroprogestinics and to follow up the patient. Since after three months we did not obtain any clinical response (persistence of primary amenorrhea), we reevaluated the patient to reestablish the correct diagnosis.
5. Balsamo et al. [1] arbitrarily stated that we reported “absence of Leydig cells” in the histological examination of the removed gonads. We are really surprised because this statement is not reported in our paper.
6. We did not perform any genetic investigations; however, we strongly agree with Balsamo et al. [1] on the importance of them in the differential diagnosis of 46,XY disorders of sex development (DSD).
7. As evidenced in many papers [5–10], the reason for postpubertal gonadectomy is the already well-established risk of testicular malignancy, which seldom occurs before puberty in CAIS. Even if we agree with Balsamo et al.'s suggestion [1] to postpone surgery until the legal age at which the propositus can participate in the decision making process, this could be considered just a delayed decision of an often necessary treatment. Moreover, all the authors of the paper “*Complete androgen insensitivity syndrome: a rare case of disorder of sex development*” [2] currently work in Italy and operate under the Italian law, as Balsamo et al. [1] do. According to the Italian law, the decision regarding disclosure of the diagnosis and medical/surgical treatment in minor patients must be demanded from their parents, unless there is a different decision from a judge. So we just followed the law of the country in which we live and operate as MD, without applying any personal opinion about it.

Once again, we thank the authors for their precious suggestions and the editor for giving us an opportunity to clarify the issues.
